# 外周血免疫指标在肺癌患者免疫治疗中的预测价值

**DOI:** 10.3779/j.issn.1009-3419.2023.102.38

**Published:** 2023-12-20

**Authors:** Shu SU, Xin LV, Liang QI, Min WEI, Baorui LIU, Lifeng WANG

**Affiliations:** ^1^210008 南京，南京大学医学院附属鼓楼医院肿瘤中心（苏舒，吕昕，祁亮，刘宝瑞，王立峰）; ^1^The Comprehensive Cancer Center of Nanjing Drum Tower Hospital, Affiated Hospital of Medical School, Nanjing University & Clinical Center Institute of Nanjing University; ^2^南京中医药大学鼓楼临床医学院（韦敏）; ^2^Nanjing Drum Tower Hospital Clinical College of Nanjing University of Chinese Medicine, 210008 Nanjing, China

**Keywords:** 肺肿瘤, 免疫治疗, 生物标志物, 疗效预测, Lung neoplasms, Immunotherapy, Biomarkers, Efficacy prediction

## Abstract

**背景与目的** 免疫治疗的应用大大改善了肺癌患者的临床结局。很大一部分肺癌患者能从程序性死亡受体1（programmed cell death 1, PD-1）/程序性死亡配体1（programmed cell death ligand 1, PD-L1）单抗治疗中获益，然而，仍有一部分耐药患者疗效不佳，临床上迫切需要能早期、便捷识别获益人群的生物标志物。在此，本研究回顾性分析了PD-1抗体治疗对局部晚期无法手术或转移性肺癌患者的疗效，并初步探索了外周血免疫指标与患者临床反应的相关性。**方法** 该单中心研究共纳入2020年3月至2021年12月接受PD-1/PD-L1抗体治疗的IIIA-IV期肺癌患者61例，收集PD-1/PD-L1抗体一线或后线治疗的病历数据。检测并分析患者外周血血清中多重Th1和Th2细胞因子水平以及外周血T细胞表型，探索细胞因子水平、T细胞表型等和患者临床反应之间的关系。**结果** 入组患者均完成至少2个周期PD-1/PD-L1单抗治疗。其中，42例（68.9%）患者获得部分缓解（partial response, PR），7例（11.5%）患者疾病稳定（stable disease, SD），12例（19.7%）患者疾病进展（progressive disease, PD）。治疗前，疾病控制（disease control rate, DCR）组（有效组）患者外周血干扰素γ（interferon gamma, IFN-γ）（P=0.023）、肿瘤坏死因子α（tumor necrosis factor α, TNF-α）（P=0.007）和白介素5（interleukin 5, IL-5）（P=0.002）水平高于PD组（无效组）患者。此外，PD-1/PD-L1抗体治疗后外周血淋巴细胞绝对计数的减少与疾病进展有关联（P=0.023）。治疗后血清中IL-5（P=0.0027）和IL-10（P=0.0208）水平较治疗前明显升高。**结论** 肺癌患者外周血血清IFN-γ、TNF-α和IL-5水平在预测抗PD-1阻断治疗临床疗效方面有一定的作用，肺癌患者外周血淋巴细胞绝对计数的减少与疾病进展有一定相关性，还需要大型前瞻性研究进一步阐明这些生物标志物的价值。

## Abstract (en)

**Background and objective** The application of programmed cell death 1 (PD-1)/programmed cell death ligand 1 (PD-L1) antibodies has greatly improved the clinical outcomes of lung cancer patients. Here, we retrospectively analyzed the efficacy of PD-1 antibody therapy in locally advanced non-surgical or metastatic lung cancer patients, and preliminarily explored the correlation between peripheral blood biomarkers and clinical responses. **Methods** We conducted a single center study that included 61 IIIA-IV lung cancer patients who received PD-1 antibody treatment from March 2020 to December 2021, and collected the medical record data on PD-1 antibody first-line or second-line treatment. The levels of multiple Th1 and Th2 cytokines in the patient's peripheral blood serum, as well as the phenotype of peripheral blood T cells, were detected and analyzed. **Results** All the patients completed at least 2 cycles of PD-1 monoclonal antibody treatment. Among them, 42 patients (68.9%) achieved partial response (PR); 7 patients (11.5%) had stable disease (SD); and 12 patients (19.7%) had progressive disease (PD). The levels of peripheral blood interferon gamma (IFN-γ) (P=0.023), tumor necrosis factor α (TNF-α) (P=0.007) and interleukin 5 (IL-5) (P=0.002) before treatment were higher in patients of the disease control rate (DCR) (PR+SD) group than in the PD group. In addition, the decrease in absolute peripheral blood lymphocyte count after PD-1 antibody treatment was associated with disease progression (P=0.023). Moreover, the levels of IL-5 (P=0.0027) and IL-10 (P=0.0208) in the blood serum after immunotherapy were significantly increased compared to baseline. **Conclusion** Peripheral blood serum IFN-γ, TNF-α and IL-5 in lung cancer patients have certain roles in predicting the clinical efficacy of anti-PD-1 therapy. The decrease in absolute peripheral blood lymphocyte count in lung cancer patients is related to disease progression, but large-scale prospective studies are needed to further elucidate the value of these biomarkers.

肺癌是全世界癌症相关死亡的主要原因，每年全球约有160万人死于肺癌^[1,2]^。根据病理类型，肺癌主要分为两种亚型：非小细胞肺癌（non-small cell lung cancer, NSCLC）（占80%-85%）和小细胞肺癌（small cell lung cancer, SCLC）。临床研究^[3,4]^表明，晚期肺癌患者接受程序性死亡受体1（programmed cell death 1, PD-1）或程序性死亡配体1（programmed cell death ligand 1, PD-L1）抗体治疗可改善临床结局，与单纯化疗相比，可改善临床反应率、生存率，并减少治疗相关的不良反应。近十年来，免疫检查点抑制剂（immune checkpoint inhibitors, ICIs）已成为治疗肺癌特别是NSCLC的标准治疗方式。然而，多项抗PD-1治疗的临床试验观察结果^[5⇓-7]^也表明，无论PD-L1表达如何，仅有约15%-25%的肺癌患者对抗PD-1单抗单药治疗有反应。

有一些预测性生物标志物有助于发现可能通过PD-1/PD-L1免疫治疗获益的肿瘤患者。已有研究^[8]^证明生物标志物PD-L1以及肿瘤突变负荷（tumor mutational burden, TMB）是肿瘤免疫治疗的预测性标志物。然而，在NSCLC中，肿瘤组织中表达的PD-L1是迄今为止唯一被认可的疗效预测因子^[9]^。这对于选择有治疗反应的患者方面具有很大限制，临床实践中在PD-L1表达阴性的情况下也有很大一部分患者对ICIs有反应。除此之外，大多数患者将同时或序贯接受包括化疗、放疗及靶向治疗等在内的多种治疗，因此PD-L1的表达模式也可能受到这些因素的极大影响。目前，包括TMB、肿瘤微环境、组织基因表达谱^[10]^等在内的其他预测性生物标志物仍在研究中，然而，这些与肿瘤组织相关的标志物成本高昂且需通过有创操作获取肿瘤组织，实践具有一定难度。因而，迫切需要能更便捷获取的外周血生物标志物来帮助识别有反应的肺癌患者。前期有研究^[11,12]^表明，一部分外周血标志物可能是抗PD-1/PD-L1治疗的潜在预后因素，包括血清标志物，如中性粒细胞-淋巴细胞比率（neutrophil-lymphocyte ratio, NLR）、血小板-淋巴细胞比率（platelet-lymphocyte ratio, PLR）、中性粒细胞计数、单核细胞绝对计数（absolute monocyte count, AMC）、嗜酸性粒细胞计数（absolute eosinophil count, AEC）或血清生物标志物如乳酸脱氢酶（lactate dehydrogenase, LDH）、血浆白蛋白（albumin, ALB）或C反应蛋白（C reactive protein, CRP）。此外，外周血PD-1^+ ^T细胞的T细胞受体（T cell receptors, TCR）谱多样性预测了癌症患者免疫治疗疗效^[13]^。有研究^[14]^还表明，治疗前外周血血清白介素（interleukin, IL）等细胞因子水平也可能是预测抗PD-1治疗肿瘤患者的临床疗效和生存率的有效指标。

本研究旨在探索与肺癌患者免疫治疗临床反应相关的外周血免疫指标，从而更便捷、高效地发现能通过PD-1/PD-L1抑制剂治疗获益的肺癌人群。

## 1 资料和方法

### 1.1 患者资料

本项单中心研究纳入了61例2020年3月至2021年12月在南京大学附属鼓楼医院肿瘤中心治疗的肺癌患者。患者平均年龄为60岁，男性46例，女性15例。纳入标准：（1）美国东部肿瘤协作组（Eastern Cooperative Oncology Group, ECOG）评分为0-2分，免疫治疗周期数≥2个；（2）IIIA-IV期不可切除非鳞状NSCLC患者、鳞状NSCLC和SCLC患者；（3）自愿接受免疫治疗前后的血液采集，用于血清细胞因子检测和T细胞表型分析。排除标准为活动性自身免疫性疾病、严重感染性疾病或全身免疫抑制使用期间。患者基线特征见表1。该回顾性观察研究经过鼓楼医院研究伦理委员会批准通过。

**表1:** 患者基线特征以及临床应答情况（n=61）

Baseline characteristics	n	Percentage
Age (yr)		
Median	60	
<70	50	82.0%
≥70	11	18.0%
Gender		
Male	46	75.4%
Female	15	24.6%
Smoking status		
Non-smoker	15	24.6%
Smoker	46	75.4%
Pathology		
Non squamous/non-small cell	40	65.6%
Squamous cell	15	24.6%
Small cell	6	9.8%
Treatment lines		
1^st^	40	65.6%
≥2^nd^	21	34.4%
Stage		
III	13	21.3%
IV	48	78.7%
Mutation status		
EGFR mutation	8	13.1%
RET mutation	1	1.6%
Non-driver mutation	52	85.2%
ECOG PS score		
0-1	37	60.7%
≥2	24	39.3%
irAEs (all stage)		
Yes	10	16.4%
No	51	83.6%
Clinical response		
PR	42	68.9%
SD	7	11.5%
PD	12	19.7%

EGFR: epidermal growth factor receptor; EOCG PS: Eastern Cooperative Oncology Group performance status; irAEs: immune-related adverse events; PR: partial response; SD: stable disease; PD: progressive disease.

### 1.2 治疗和研究评估

患者每3周接受含铂标准方案的化疗，包括白蛋白紫杉醇（260 mg/m^2^）或培美曲塞（500 mg/m^2^）联合卡铂[曲线下面积（area under the curve, AUC）=5]、洛铂（30 mg/m^2^）或顺铂（75 mg/m^2^）以及PD-1/PD-L1抗体（帕博利珠单抗200 mg、纳武利尤单抗3 mg/kg、卡瑞利珠单抗200 mg、信迪利单抗200 mg、替雷利珠单抗200 mg、阿特珠单抗1200 mg、度伐利尤单抗1200 mg）治疗。对于维持治疗，根据病理表型，患者使用PD-1/PD-L1抗体治疗，使用或不使用白蛋白紫杉醇或培美曲塞维持。根据实体瘤疗效评价标准（Response Evaluation Criteria in Solid Tumors, RECIST）1.1版，每2个治疗周期评估一次临床疗效。标准为：（1）完全缓解（complete response, CR）：靶病灶完全消退；（2）部分缓解（partial response, PR）：总靶病灶减少30%以上；（3）疾病稳定（stable disease, SD）：总靶病灶减少少于30%或增加少于20%；（4）疾病进展（progressive disease, PD）：总靶病灶增加20%以上；（5）疾病控制率（disease control rate, DCR）：临床应答或稳定的比例（CR+PR+SD）。

### 1.3 样品采集和流式细胞术

在首次影像评估的同时采集61例肺癌患者PD-1/PD-L1抗体治疗前后的外周血，进行外周血单个核细胞（peripheral blood mononuclear cell, PBMC）分离和血清的分离，后续进行细胞因子检测以及表面分子流式细胞检测。选择淋巴细胞分泌上清中Th1以及Th2型细胞因子，代表细胞免疫应答以及体液免疫应答。具体实验方法如下：在免疫治疗前一天收集10 mL血液样本，然后在2 h内将样品转移到实验室进行分离。1200 rpm离心7 min后收集上清，加入抗体偶联beads制备混合样品溶液，采用CBA（Cytometric Bead Array, Biolegend, San Diego, CA, USA）分析试剂盒检测IL-2、IL-4、IL-5、IL-6、IL-10、IL-13、干扰素γ（interferon gamma, IFN-γ）和肿瘤坏死因子α（tumor necrosis factor α, TNF-α）。细胞沉淀采用PBS重悬加入Ficoll Plaque（GE, Chicago, IL, USA）离心纯化，制备PBMC悬液，并用CD3、CD4、CD8、CD274、CD279、TIGT、CD27、CD11b、CD33、CD45RO、CD62L、CD127和CD38（Biolegend, San Diego, CA, USA）特异性的荧光流式抗体标记以进行流式细胞分析。

### 1.4 统计分析

使用GraphPad Prism 9软件和R语言进行统计分析。通过t检验以及非参数Wilcoxon检验分析细胞因子水平、T细胞表型和临床应答之间的统计学差异。P<0.05为差异有统计学意义。

## 2 结果

### 2.1 患者血清IFN-γ、TNF-α和IL-5水平对抗PD-1治疗的预测价值

收集61例接受PD-1或PD-L1单抗治疗的晚期肺癌患者治疗前的血清，对IL-2、IL-4、IL-5、IL-6、IL-10、IL-13、IFN-γ和TNF-α进行检测和分析。根据DCR中临床疗效的定义，PR+SD（n=49）定义为有效，PD定义为无效（n=12）。治疗前外周血中IFN-γ（P=0.023）、TNF-α（P=0.007）和IL-5（P=0.002）水平DCR组高于PD组。而两组间血清中的其余细胞因子IL-2、IL-4、IL-6、IL-10和IL-13水平与PD-1或PD-L1单抗治疗的疗效差异无统计学意义（P>0.05）（图1）。这些结果表明，肺癌患者体内预先存在的较高水平的Th1型细胞因子，如IFN-γ和TNF-α以及Th2型细胞因子IL-5，可能是预测PD-L1/PD-1阻断治疗获益的良好指标。

**图1:**
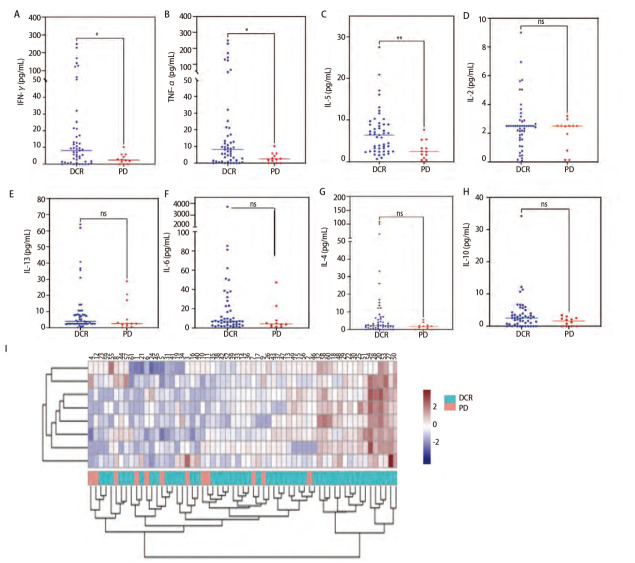
治疗基线外周血清细胞因子水平情况与临床应答相关性。A-H：肺癌患者免疫治疗基线外周血细胞因子分泌水平情况及与临床应答相关性；I: 肺癌患者免疫治疗基线外周血细胞因子分泌水平在各疗效组人群中的热图分析。

### 2.2 治疗后血清IL-5和IL-10水平升高提示抗PD-1治疗临床获益

通过评估Th1/Th2细胞因子，包括IL-2、IL-4、IL-5、IL-6、IL-10、IL-13、IFN-γ和TNF-α在治疗前后表达变化情况表明，在有效组（DCR）的患者中，免疫治疗后血清中IL-5（P=0.0027）和IL-10（P=0.0208）水平较治疗前明显升高。然而，其他细胞因子，包括IFN-γ（P=0.1775）和TNF-α（P=0.1884），在DCR组患者中，治疗后表达水平没有显著上调。同样，IL-2（P=0.1300）、IL-4（P=0.1829）、IL-6（P=0.4911）和IL-13（P=0.0879）在DCR组患者血清中并没有检测到显著的变化（图2）。

**图2:**
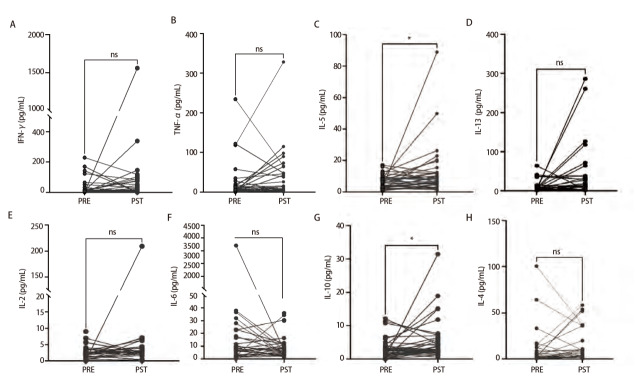
疾病控制组肺癌患者免疫治疗前后外周血清细胞因子水平变化情况。A：治疗前后IFN-γ水平情况；B：治疗前后TNF-α水平情况；C：治疗前后IL-5水平情况；D：治疗前后IL-13水平情况；E：治疗前后IL-2水平情况；F：治疗前后IL-6水平情况；G：治疗前后IL-10水平情况；H：治疗前后IL-4水平情况。

### 2.3 外周血淋巴细胞绝对计数的减少与抗PD-1阻断的疾病进展相关

在DCR组队列（n=49）中，治疗前后淋巴细胞绝对计数没有差异（P=0.2911）。而在PD组（n=12）治疗后，淋巴细胞绝对计数显著降低（P=0.023）（图3）。这些结果表明，PD-1抗体治疗后外周血淋巴细胞绝对计数的减少与疾病进展相关。这一发现也有助于在影像评估前尽早识别未能从免疫治疗中获益的患者。

**图3:**
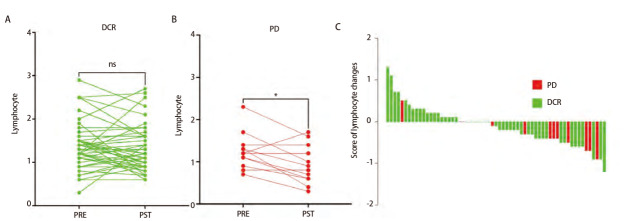
治疗前后外周血淋巴细胞计数变化情况。A: 疾病控制组治疗前后淋巴细胞总计数情况；B: 疾病进展组治疗前后淋巴细胞总计数情况；C：不同疗效组淋巴细胞总计数变化情况瀑布图。

### 2.4 抗PD-1阻断剂对T细胞表型和临床反应的预测价值

对61例肺癌患者在接受免疫治疗基线时PBMC中的CD4^+^和CD8^+ ^T细胞各亚群进行了流式细胞分析。通过分离PBMC并用流式抗体染色，包括活化和共刺激分子标志物（如CD27、CD38）、抑制性分子标志物（PD-1、PD-L1、TIGT）、调节性T细胞标志物（CD25、CD127）以及记忆和效应标志物（CD45RO、CD62L）评估DCR组和PD组患者T细胞的基线表型特征是否不同。如图4所示，两组间CD4^+ ^T细胞或CD8^+ ^T细胞上的大多数标志物没有统计学差异。在DCR组中，CD4^+ ^T细胞上CD45RO^+^CD62L^+^（中央记忆表型）/CD45RO^+^CD22L^-^（效应记忆表型）的比率有高于PD组的趋势，但无统计学差异（P=0.23）。除此之外，通过分析T细胞表面活化和共刺激标志物发现CD8^+^CD27^+ ^T细胞的百分比较高与临床反应答较差有关联（P=0.0096）。然而，基线CD4^+ ^T细胞或CD8^+ ^T细胞上抑制性分子标志物PD-1、PD-L1或TIGT在DCR组和PD组的患者之间并未显示出统计学差异。这些结果也表明，使用T细胞特定表型来确定PD-1/PD-L1抑制剂对肺癌患者的预后仍极具挑战。

**图4:**
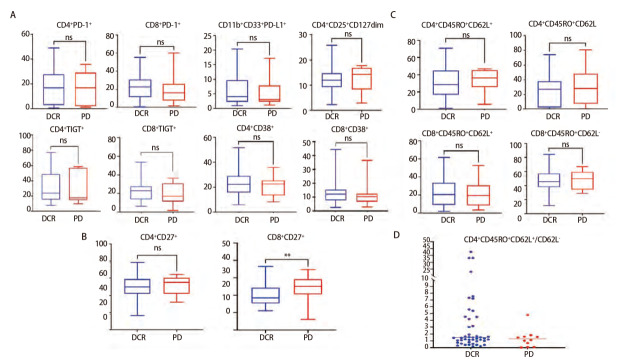
不同疗效组免疫治疗基线外周血T细胞亚群组成情况。A：CD4^+ ^T细胞以及CD8^+ ^T细胞表面各亚群表达情况；B：CD4^+ ^T细胞以及CD8^+ ^T细胞CD27分子表达情况；C：CD4^+ ^T细胞以及CD8^+ ^T细胞记忆以及效应亚群表达情况；D：CD4^+^中央记忆性T细胞与效应记忆性T细胞比例。

## 3 讨论

近年来，免疫疗法在肺癌患者的治疗中得到了广泛的应用。然而，仍有一些患者对免疫治疗存在原发性耐药，或者在免疫治疗过程中出现获得性耐药。PD-1阻断疗法的耐药机制尚未得到很好的阐明，可能涉及多种因素，包括异常肠道微生物组成以及抗原呈递功功能的缺失等。因而，需要有生物标志物以便能有效地预测肺癌患者对PD-1阻断治疗的疗效^[15⇓-17]^。肿瘤组织PD-L1的表达强弱是目前已明确的NSCLC免疫治疗的预后因素。除外PD-L1，其他一些分子标志物包括TMB、肿瘤微环境、组织基因表达谱等仍在研究中，然而，这些标志物需通过有创操作获取肿瘤组织，成本较高且在动态监测方面具有一定的局限性。因此，迫切需要外周血生物标志物以便能更快捷、有效地预测肺癌患者对PD-1阻断治疗的疗效。

本研究结果显示，肺癌患者外周血中预先存在较高水平的Th1细胞因子，如IFN-γ、TNF-α和Th2细胞因子IL-5，可能提示对PD-L1/PD-1阻断治疗有更好的临床应答。从机制上来说IFN-γ是通过增强自然杀伤细胞以及T细胞的细胞毒性和增强树突状细胞功能等几方面来调节天然免疫应答和适应性免疫应答来增强患者抗肿瘤免疫反应^[18]^。同样，TNF-α在肿瘤微环境中通过招募和激活中性粒细胞、巨噬细胞和淋巴细胞，从而发挥抗肿瘤免疫应答的作用^[19]^。既往有研究^[20]^表明在免疫治疗前和治疗开始后3个月，IFN-γ和TNF-α细胞因子水平的增加与NSCLC免疫治疗反应的改善和总生存期（overall survival, OS）延长有关。而本研究结果表明，预先存在的细胞免疫应答可能有助于PD-1抗体发挥较强和持久的免疫反应。肿瘤免疫主要由Th1介导的细胞免疫介导，这能解释为什么较高的IFN-γ和TNF-α细胞因子水平与更好的预后相关。同时，本研究也观察到，预先存在较高水平的IL-5与更好的临床应答相关。IL-5主要由Th2淋巴细胞和2型固有淋巴细胞（group 2 innate lymphoid cell, ILC2）产生。它可以通过促进B细胞的分化和生长来增加抗体分泌，并增强支持抗体应答的体液免疫应答^[21]^，从而更好地协同细胞免疫应答发挥作用。本研究还发现治疗后患者血清IL-5和IL-10水平的升高提示与抗PD-1单克隆抗体治疗的疗效有关。这可能提示，细胞因子水平的动态变化与治疗的客观反应率也有一定的关联。此外，研究还观察到在免疫治疗过程中肿瘤进展患者的总淋巴细胞绝对计数明显减少， 这也有助于我们早在影像评估之前识别那些未能从免疫治疗中获益的人群。

肿瘤微环境中的CD8^+ ^T细胞和CD4^+ ^T细胞免疫在介导持久抗肿瘤反应中发挥重要作用。研究^[22]^表明，外周血中的中央记忆CD4^+ ^T细胞可以作为恶性黑色素瘤患者对PD-1阻断治疗临床反应的预测指标。本研究观察到CD4^+ ^T细胞上CD45RO^+^CD62L^+^（中央记忆表型）/CD45RO^+^CD62L^-^（效应记忆表型）比率较高的患者与PD-1阻断的临床疗效有一定关联，但无明显差异，这可能是由于样本数相对较少、数据偏倚等所致，后续需要更大样本量的验证。此前的研究表明，CD27配体CD70表达于树突状细胞表面，是CD8^+ ^T细胞有效活化和发挥抗肿瘤免疫应答所必需的^[23⇓⇓⇓⇓-28]^。然而，本研究基线时外周血中CD8^+^CD27^+ ^T细胞比例与临床反应呈负相关。但事实上，之前有研究^[29]^通过供体淋巴细胞过继输注的动物模型发现CD8^+ ^T细胞因持续暴露于抗原后因活化而耗尽，通过激动剂抗CD27和抗PD-L1的组合可以实现短暂的CD8^+ ^T淋巴细胞功能恢复。这可能是耗竭的CD8^+^CD27^+^细胞对PD-1阻断缺乏应答的原因之一。

有研究^[30⇓⇓⇓-34]^表明在CD8^+ ^T细胞表面未检测到明显的与治疗或应答相关表型差异，包括PD-1以及PD-L1，这和本研究结果相一致。然而，也有研究^[35]^表明，PD-1阻断治疗后PD-1^+^CD8^+^ T细胞的早期增殖与临床应答相关，外周血细胞上PD-1的表达可能提示肿瘤反应性T细胞的富集。针对PD-L1在外周血细胞上的表达，有一项对32例接受PD-L1/PD-1阻断治疗的NSCLC患者的研究结果^[36]^提示在应答者和无应答者之间PD-L1^+^CD11b^+^髓系细胞群体的百分比存在显著差异，基线时PD-L1^+^CD11b^+^细胞百分比>30%的患者显示出更高的临床应答率。然而，本研究并未观察到外周血中CD33^+^CD11b^+^PD-L1^+^细胞的表达对PD-1/PD-L1阻断治疗有预测价值，这也提示在分析外周血中的这些标志物时，应考虑比肿瘤环境更复杂的详细的分子标志物组合以及门控分析策略。此外，除了PD-1或PD-L1细胞的绝对数量外，肿瘤抗原特异性免疫细胞的TCR多样性和特异性也可能在预测中发挥重要作用。已有研究^[31,37,38]^表明，外周PD-1^+^CD8^+ ^T细胞的TCR多样性和新抗原特异性CD8^+ ^T细胞在预测抗PD-1/PD-L1对于NSCLC治疗的临床疗效方面有一定价值，这也为我们后续进一步的深入研究提供了一定的思路。

本研究有几个局限性。首先，这是一项回顾性的单中心研究，入选的患者数量相对较少，治疗前后配对的患者样本量有限，并且入组患者包括鳞癌、腺癌、SCLC等多种肺癌亚型，其生物学特性有着一定差异，未进一步分析血清指标的变化和不同病理亚型的肺癌疗效之间的相关性，我们在后续的研究中也将进一步扩大样本量以及进行亚组分析进一步明确；第二，本研究将所有接受PD-1/PD-L1抗体治疗的患者纳入研究，其中包括大部分联合化疗的患者，但并没有设立仅接受化疗的对照组。因而，细胞因子水平或免疫细胞表型可能受化疗药物的影响，并不能纯粹归因于免疫治疗；第三，这些参数的截断（cut-off）值没有进一步确定，不同的cut-off值可能会对结果产生影响；第四，该研究包括了一部分SCLC患者，其生物学性质以及对免疫治疗的应答模式与NSCLC也有着较大差异，可能会对研究结果造成一定影响和偏倚；第五，在这项研究中，1/3的患者作为二线或二线后治疗接受PD-1抑制剂，因而基线血液标志物可能受到先前治疗（包括化疗、靶向或其他治疗）的影响。尽管存在这些局限性，这项研究仍是首次确定晚期肺癌患者基线Th1/Th2细胞因子水平和抗PD-1/PD-L1阻断性抗体治疗疗效有关，并初步探索了这些细胞因子在外周血的动态变化趋势与免疫治疗疗效的关系。

总之，本研究初步表明，肺癌患者基线血清IFN-γ、TNF-α和IL-5水平可以预测抗PD-1阻断治疗的临床疗效，且治疗后IL-5和IL-10的上调可能预示更好的临床应答，而免疫治疗后外周血总淋巴细胞计数的减少可能预示预后的不佳。此外，CD4^+^CD45RO^+^CD62L^+^/CD4^+^CD45RO^+^CD62L^-^亚群的比率较高似乎与较好的临床应答有关。大型前瞻性研究有待进一步阐明这些生物标志物的价值。相信这些外周血生物标志物可以帮助临床中更早期识别能够从抗PD-1/PD-L1免疫治疗中获益的优势人群。

**Competing interests**

The authors declare that they have no competing interests.

**Author contributions**

Wang LF and Liu BR conceived and designed the study. Su S, Lv X and Wei M performed the experiments and analyzed the data. Qi L contributed analysis tools. All the authors had access to the data. All authors read and approved the final manuscript as submitted.
